# MgSO_4_ as a novel hypothermia infusion solution promotes ischemic stroke recovery through Ca^2+^ regulation of neurovascular units

**DOI:** 10.7150/thno.104879

**Published:** 2025-01-02

**Authors:** Yang Zhang, Miaowen Jiang, Ming Wei, Chuanjie Wu, Yiming Huang, Baoying Song, Yi Xu, Hongkang Zhang, Yunong Shen, Di Wu, Yufeng Zheng, Ming Li, Xunming Ji

**Affiliations:** 1 Department of Neurology, Xuanwu Hospital, Capital Medical University, Beijing, China.; 2 China-America Institute of Neurology, Xuanwu Hospital, Capital Medical University, Beijing, China.; 3 Beijing Institute of Brain Disorders, Capital Medical University, Beijing, China.; 4 Department of Neurosurgery, Tianjin Huanhu Hospital, Tianjin, China.; 5 School of Materials Science and Engineering, Peking University; Beijing, China.; 6 Department of Neurosurgery, Xuanwu Hospital, Capital Medical University, Beijing, China.

**Keywords:** magnesium sulfate, hypothermia, ischemic stroke, neurovascular units, calcium

## Abstract

The neuroprotection of acute ischemic stroke (AIS) patients to alleviate the reperfusion injury is a long-standing problem. Although the intra-arterial selective cooling saline infusion (IA-SCSI) is a promising technique to improve the outcome of AIS, the clinical results are not sufficiently satisfactory. Magnesium-containing solutions such as magnesium sulfate (MgSO_4_) can protect cells by modulating the intracellular Ca^2+^ content in damaged cells, suggesting that they could be hypothermia infusion solution. In this study, we hypothesized that MgSO_4_, as a novel hypothermia infusion solution (IA-SCMI), can promote AIS recovery.

**Methods:** First, IA-SCMI was performed in rats subjected to the middle cerebral artery occlusion. The effect of combined hypothermia and MgSO_4_ treatment on different neurovascular (NVU) cells after oxygen-glucose deprivation/reoxygenation (OGD/R) injury was examined.

**Results:** Compared with the IA-SCSI or IA-MgSO_4_ infusion alone, IA-SCMI offered the best protective effect by improving neurological defects, and alleviating brain integrity damage and CBF reduction. Meanwhile, *in vitro* results revealed that the combination of hypothermia and MgSO_4_ provided maximum protection to the NVU cells, and this protective effect was mainly achieved through the regulation of Ca^2+^ homeostasis in different cells.

**Conclusion:** MgSO_4_ may be a promising hypothermia infusion solution to facilitate the recovery of neurological function in AIS patients.

## Introduction

Acute ischemic stroke (AIS) is the leading cause of morbidity and mortality worldwide 1, 2. The validation and widespread adoption of thrombectomy and thrombolysis have ushered in the era of reperfusion therapy for AIS 3. However, nearly half of patients with complete proximal large artery recanalization do not achieve functional independence, due to potential ischemia/reperfusion (I/R) injury 4. Therefore, it is essential to develop alternative strategies in combination with successful recanalization to enhance patient outcomes.

Hypothermia therapy is a long-standing method for treating brain injury including ischemic stroke. It confers neuroprotective effects by modulating diverse neurovascular units (NVUs) 5, 6 such as neural protection and blood-brain barrier (BBB) restoration. Owing to its serious systemic side effects 7, whole body hypothermia is increasingly being replaced by selective brain cooling like ice cap cooling 8 and transnasal cooling 9. Furthermore, advances in endovascular treatment have led to the development of a more precise technique, intra-arterial selective cooling saline infusion (IA-SCSI) 10, 11. This method has been validated in AIS model of various animals, such as rats 12 and rhesus monkeys 13. However, the clinical outcomes are not sufficiently satisfactory 14. Besides, hypothermia has been indicated to reduces cerebral capillary blood flow, thus maybe hinder microcirculation reperfusion after recanalization 15. Therefore, it is imperative to formulate new perfusion solutions that can replace saline to facilitate neuroprotection and prevent the microcirculation damage induced by hypothermia. Magnesium-containing solutions seem to be a good candidate, with previous studies showing that surface hypothermia combined with intravenous infusion of Mg^2+^ promotes AIS recovery 16, 17.

Magnesium-containing solutions like magnesium sulfate (MgSO_4_) and magnesium chloride (MgCl_2_) exert multiple neuroprotective effects 18. Briefly, Mg^2+^ as an endogenous antagonist of the calcium channel might decrease brain excitotoxicity and cell death induced by calcium overload following nervous system injury 19. Studies have demonstrated that Mg^2+^ affects the permeability of the BBB and inhibits inflammatory response 20, 21. Moreover, Mg^2+^ could block calcium channels to prevent the synthesis of endothelin-1, inhibit myosin light chain kinase activation in vascular diameter regulating cells and attenuate vasospasm and contraction, leading to increased cerebral blood flow 22. Therefore, Mg^2+^-containing solutions, with multiple NVU regulatory effects on calcium, seems to be a good choice for intra-arterial selective cooling infusion. Furthermore, considering that hypothermia therapy can regulate calcium channels 23, 24, the respective co-regulatory effect of the combination of hypothermia and Mg^2+^ therapy on the calcium ion of different NVU cells should be focused on, which has never been investigated before because most predecessors only observed the impact of hypothermia or Mg^2+^ on a single type of cell.

In this study, we used MgSO_4_ as novel intra-arterial selective cooling infusion solution (IA-SCMI) to determine whether this therapeutic strategy could provide additional neuroprotection. To test this hypothesis, IA-SCMI was performed in rats subjected to transient middle cerebral artery occlusion (MCAO) and the behaviors of rats were tested. In addition, the degree of neuron damage, BBB breakdown and cerebral blood reflow (CBF) was evaluated. Finally, oxygen-glucose deprivation/reoxygenation (OGD/R) models were established *in vitro* for different NVU member cells (neurons, microvessel endothelial cells (ECs) and pericytes) which were then treated with hypothermia and Mg^2+^ to investigate the multiple cerebral protective mechanisms of the combination therapy, especially from the perspective of calcium ion regulation.

## Methods

### Animal and live experimental process

Male mature Sprague-Dawley (SD) rats weighing between 280 and 320 g were used in all experiments. All experimental procedures were approved by Institutional Animal Investigation Committee of Capital Medical University. The animal treatments were conducted following the National Institutes of Health's guidelines for the Care and Use of Laboratory Animals.

**Figure 1:**
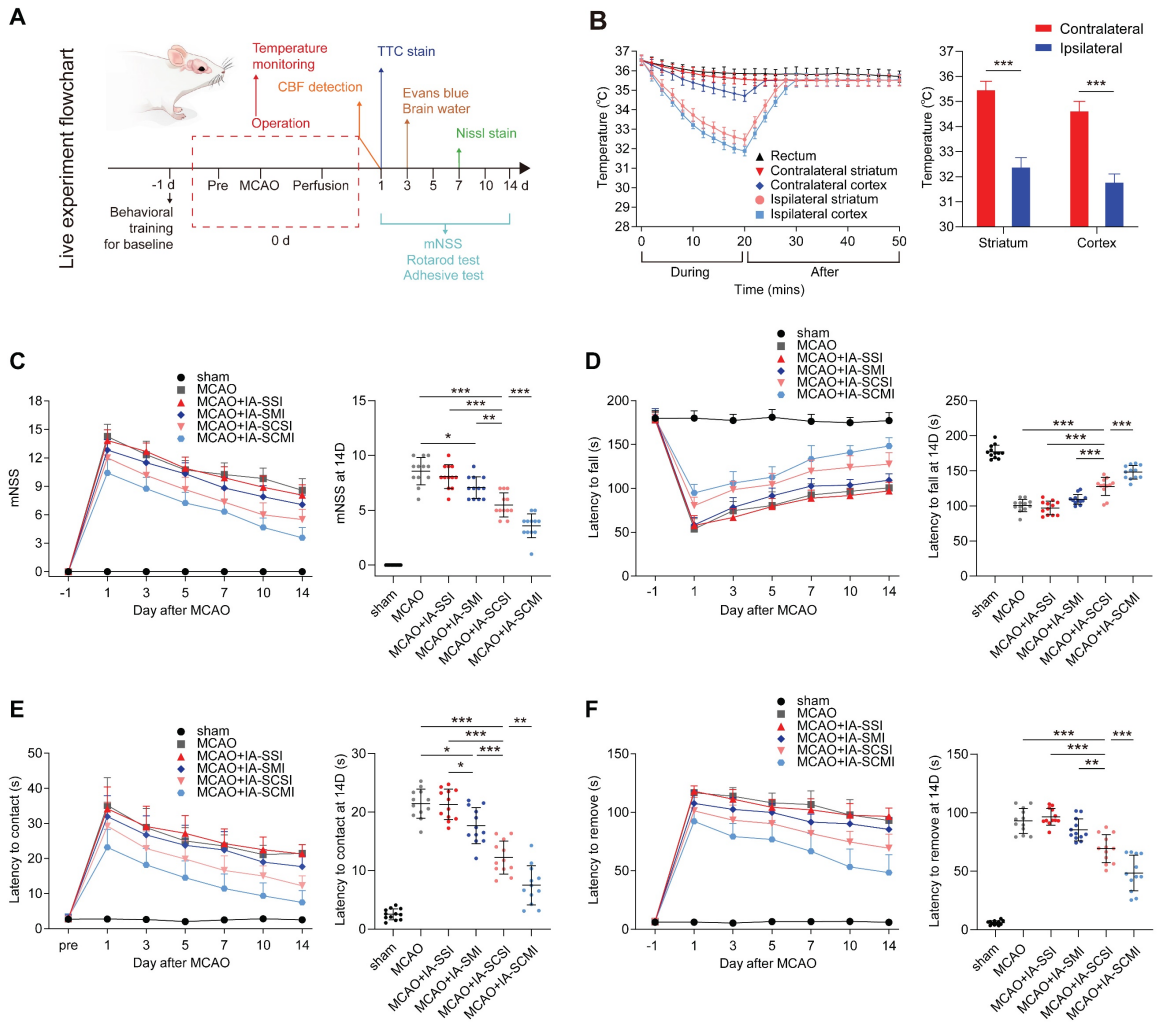
The *in vivo* cooling and neurological protection effect of IA-SCMI. (A) The flow chart of the live experiment. (B) The cooling effect of IA on the ipsilateral and contralateral cortex and striatum (n=5). (C) mNSS score (n=12). (D) Rotarod test (n=12). (E-F) Adhesive removal test (n=12). * *p* < 0.05, ** *p* < 0.01, *** *p* < 0.001.

Procedurally, the cooling effect of IA-SCMI was explored *in vivo*, and the live experiments were divided into two parts: behavioral testing and histological evaluation. Behavioral tests included the modified neuropathy symptom score (mNSS), rotarod test, and adhesive removal test. The rats were trained one day before surgery to obtain baseline scores, and then tested on day 1, 3, 5, 7, 10, and 14 after operation. All tests were supervised by the same person, who was blinded to the treatment groups. Histological evaluation was conducted to assess neural integrity, BBB damage, and CBF. Neural cell damage was evaluated through short-term (one day post-surgery) assessment of cerebral infarction volume using triphenyltetrazolium chloride (TTC) staining and long-term evaluation of neural damage using Nissl staining seven days post-surgery. BBB damage was assessed on the third day post-surgery using Evans blue (EB) staining and brain water content measurement. CBF was continuously monitored before and during the MCAO, and within one day after reperfusion. The procedure of live experiments is shown in Figure 1A.

### MCAO model

To mimic AIS I/R injury, we employed a transient MCAO procedure, as commonly described in the literature 25. Five percent enflurane (RWD Life Science Co., LTD, China) was used for anesthesia, followed by maintenance with 1% to 3% enflurane. The skin of the rat's neck was shaved and disinfected, followed by creation of a longitudinal skin incision to expose the right common carotid artery (CCA), external carotid artery (ECA), and internal carotid artery (ICA). A tiny cut was made in the ECA, and a thread (RWD Life Science Co., LTD, China) was put through ECA and ICA to the right MCA's beginning (Figure S1A). After occlusion for 2 h, the thread was removed to achieve reperfusion of MCA.

### Intra-arterial selective infusion

Intra-arterial selective infusion (IA-SI) involved the infusion of 10 mL of different solutions, administered at the onset of reperfusion following a previously published method 26. Rats in the four groups were subjected to IA-SI as follows: normothermic saline group (IA-SSI), normothermic MgSO_4_ (3.6 mg/ml, Aladdin Biochemical Technology Co., Ltd, China) group (IA-SMI) 27, hypothermic (4℃) saline group (IA-SCSI), hypothermic MgSO_4_ group (IA-SCMI). A small incision was created in the right ECA of each rat. This incision was used to advance a modified PE-10 catheter to reach the ICA. Subsequently, the solution was infused at a constant speed for 20 min using an injection pump (Figure S1B-C). Evaluating rats' tolerance to IA-SI was based on measurements of some physiological parameters including the mean arterial blood pressure (MABP), heart rate and arterial oxygen partial pressure (pO_2_) and arterial carbon dioxide partial pressure (pCO_2_) before, during and after the IA-SI. The MABP and heart rate were recorded using an Automated Non-Invasive Blood Pressure Recording Tail-Cuff system (Softron, Japan). pO_2_ and pCO_2_ were determined using arterial blood collecting from the femoral artery of rats with a blood gas analyzer (Cornley, China).

### Brain and core temperature monitoring

Brain and rectal temperatures were continuously monitored before reperfusion, during and after IA-SI treatment using superfine temperature-sensor wires connected to a thermometer (Aidiwen Co., Ltd, China) as described previously (Figure S1C-D) 28, 29. For brain temperature, wires were placed 3 mm lateral to the bregma, 4 mm in depth (striatum temperature), and 3 mm lateral and 3 mm posterior to the bregma, 2 mm in depth (cortical temperature), respectively. The core temperature was monitored by inserting wires into the rectum. To maintain core temperature, rats were placed on a feedback-regulated heating pad (RWD Life Science Co., LTD, China) during operation.

### Neurological behavioral tests

Neurological behavioral tests, including mNSS score, rotarod test, and adhesive removal test, were performed at the same time of day to control for circadian rhythm effects on behavior. The mNSS comprised assessments of motor function, sensory function, reflexes, balance, and abnormal movements 30, with scores ranging from 0 to 18 (normal score: 0; maximal deficiency score: 18). The details of the mNSS scoring procedure are presented in Table S1. In the rotarod test 30, 31, rats were placed on an accelerating rotating rod (RWD Life Science Co., LTD, China) and their latency to fall was measured. The rod speed was gradually increased from 4 to 40 rpm over 5 minutes. For the adhesive removal test 32, a piece of sticky tape was applied to the rat's left paw, and the time taken to remove the tape was recorded. If rats were unable to remove the tape within the allotted time limit of 2 min, the removal time was recorded as 2 min.

### Infarct volume

The rats were transcardially perfused with phosphate-buffered saline (PBS) and brains were collected. Brains were cut into five 2-mm sections and stained with 2% TTC (wt/vol in PBS, Solarbio Bioscience & Technology Co., LTD, China) for 20 min at 37°C to measure the infarct volume. After capturing images of the brain slices, the infarct volume ratio was calculated using ImageJ software (National Institutes of Health, USA) using the following formula: infarct volume ratio = (contralateral hemisphere area - noninfarcted area of the ipsilateral hemisphere) / contralateral hemisphere area.

### Neuron damage

The neuron damage was evaluated by performing Nissl staining on brain slices according to previous descriptions 33, 34. The brain tissue was emersed in 10% formaldehyde for more than 24 h, and then embedded in paraffin, sectioned and stained with the Nissl method. Stained sections (corresponding to coronal slices from the rostral region (Bregma +1.24mm to -4.16 mm)) were photographed and the most severely damaged brain sections were selected for display (Bregma -2.26±0.20 mm). Sections were analyzed by Image J software to count the cells that tested positive for Nissl (clear Nissl bodies and nuclei) in both sides of the cortex and striatum. The reasons for choosing these two specific areas (cortex and striatum) for analysis were as follows: firstly, we measured these two areas during temperature measurement, so the neuron damage analysis also corresponded to these two areas; secondly, the neurofunctional assessment of the present research mainly focused on sensory and motor function, which were the main responsibility of these two areas. On each side of the cortex and striatum, five regions were randomly selected. The selected region of interest is completely symmetrical on both hemispheres. The ratio of Nissl-positive cells in the ipsilateral hemisphere to the contralateral hemisphere was calculated. A lower Nissl body ratio indicated more serious neuron damage.

### Evans blue leakage

Evans blue leakage measurement was performed according to the previously reported method 28, 35. The tail vein was punctured with a 2% Evans blue dye solution (wt/vol in PBS, 3 mL/kg, 0.84-0.96 ml due to the weight of rats ranging from 280 to 320 g, Solarbio Bioscience & Technology Co., LTD, China), and rats were killed 2 h later through cardiac perfusion with saline. The brains were then removed and photographed to visualize Evans Blue leakage. Subsequently, ischemic half-brain tissue was homogenized in one milliliter of formamide solution (Solarbio Bioscience & Technology Co., LTD, China) to create a cell suspension. After a 24-h incubation period at 60°C, the material was centrifuged for 10 min at 5000 rpm. The Evans blue content was calculated from a standard curve constructed by optical density readings of the supernatant at 635 nm using a microplate spectrophotometer (Bio-Tek, USA).

### Brain water

Fresh brain tissue was weighed to calculate the wet weight, which was then dried in an oven to obtain dry weight. Brain tissue water content percentage was calculated as (wet weight - dry weight) / wet weight × 100% 36.

### Cerebral blood flow

The relative CBF quantify was measured using a Laser speckle imaging (LSI, RWD Science Co., LTD China) following a previously published technique 37. The rats were placed on a prone position and their heads were fastened in a stereotaxic device after shaving and iodophor disinfection. A longitudinal incision was made in the skin over the cranium. The cranium, from bregma to lambda, was thinned using a dental drill under a dissecting microscope until CBF could be clearly quantified by LSI. The mean CBF of both sides was calculated and the operation ipsilateral side's CBF was expressed as a percentage of the value on the contralateral side.

### Immunofluorescence

The immunofluorescence labeling procedure was conducted as previously described 38. Briefly, the coronal brain slices (20 µm thickness) and seeded cells were incubated with the blocking solution (10% secondary antibody species serum and Triton 0.05-0.1% in PBS) for 1 h. The sections and cells were incubated overnight at 4°C with the corresponding primary antibodies diluted in the blocking solution. Sufficient secondary antibodies conjugated to fluorophores were utilized. The pericytes were also incubated with phalloidin and DAPI (Solarbio Bioscience & Technology Co., LTD, China) to visualize F-actin and cell nucleus. Finally, fluorescent images were recorded for subsequent analysis. The primary antibodies included anti-Rat CD31 (Cat#GB120005, Servicebio, China), anti-Rat Ly6G (Cat#GTX40912, GeneTex, USA), anti-Mouse ZO-1 (Cat#33-9100, Invitrogen, USA), anti-Rat α-SMA (Cat#14395-1-AP, Proteintech, USA) and anti-Rat TRPM8 (Cat#ACC-049, Alomone Labs, Israel).

### OGD/R model

Neural cells (HT22), cerebral microvessel ECs (bEnd.3) and rat cerebral primary pericytes were washed and cultured in glucose-free Dulbecco's modified Eagle's medium (DMEM, Gibco, USA) without fetal bovine serum (FBS), and subsequently moved into an anaerobic chamber containing 5% CO_2_ and 95% N_2_ for 4-hour incubation. The cells were then divided into 4 groups: OGD/R group, the cells were placed back into the normoxic incubator (37°C, 5% CO_2_) with regular media containing 10% FBS (Gibco, USA) for 24 h; OGD/R+HT group, the cells were placed in a hypothermia normoxic incubator (32°C) with regular media; OGD/R+MgSO_4_ and OGD/R+MgSO_4_+HT group were placed in 37°C and 32°C normoxic incubator, respectively, with regular media containing 10 mM MgSO_4_. A 10 mM concentration of MgSO_4_ was selected based on findings indicating its optimal neuroprotective efficacy against OGD/R-induced neuronal injury (Figure S2). Cells in the control group were cultured under normal conditions in the absence of OGD/R or other treatments for 28 h.

### Primary pericyte culture

The SD rats were euthanized through vertebrae removal, and immersed in a 75% ethanol beaker for disinfection. The brain was removed and the cerebral cortex tissue was separated and cut into 1 mm^3^ fragments. It was washed with PBS once and placed in a centrifuge tube. Subsequently, a digestion solution (collagenase II) was added for incubation at 37°C in water bath on a shaker for 1 h. The tissue suspension was centrifuged at 800 g for 5 min and resuspended to precipitate with 20% bovine serum albumin. This was followed by a further centrifugation at 1200 g for 25 min and incubation with digestive solution (dispase II) to digest at 37°C for 40 min. The solution was centrifuged at 800 g for 5 min. The precipitate was resuspended in PBS and slowly added to the upper layer of a 35% Percoll continuous density gradient cell separation solution (obtained by centrifugation at 12000 g for 1 h). The solution was then centrifuged at 1000 g for 10 min, and the yellow-white layer (microvessel) near the bottom of the red precipitate was extracted.

The microvascular segment was centrifuged at 800 g for 5 min. The cells were resuspended in a complete culture medium of rat brain microvascular pericytes, implanted into culture dishes pre-coated with polylysine, and incubated in an incubator with a constant temperature at 37°C and 5% CO_2_ for 48 h. The purity of the cells was identified by α-SMA (Figure S3).

### Normal pericyte experiment

The pericytes were divided into two groups: the MgSO_4_ group and control group. Cells in the MgSO_4_ group were treated in a medium containing 10 mM MgSO_4_. Those in the MgSO_4_ and control groups were incubated in a normoxic incubator at 32°C for 24 h, and then rewarmed to 37°C. The MgSO_4_ was added to the cells just before they were transferred to the incubator.

The pericytes were also incubated with the TRPM8 antagonist and agonist. For the antagonist tests, the pericytes were treated with 10 μM AMTB 39, and incubated in a normoxic incubator at 32°C for 24 h. The pericytes incubated without AMTB treatment served as the control. AMTB was applied just before the cells were transferred to the incubator. For the agonist tests, the pericytes were incubated with 10 mM MgSO_4_ and 0.1mM menthol in a normoxic incubator at 37°C 40. Pericytes incubated in the absence of MgSO_4_ served as the control.

### Cell viability

Cell viability was assessed using the cell counting kit-8 (CCK-8) assay and by measuring lactic dehydrogenase (LDH) release concentration. Cells were seeded at a density of 5000 cells/well in 96-well plates. For the CCK-8 assay, 10 μL of CCK-8 solution (Beyotime, China) was added to each well and incubated at 37°C for 1 h, followed by absorbance measurement at 450 nm. For LDH release measurement, a commercial LDH assay kit (Beyotime, China) was used. Briefly, supernatants collected from each well were transferred to a new 96-well plates and incubated with the reagent of assay kit at room temperature (RT) for 30 min. Finally, the absorbance was measured at 490 nm.

### Intracellular Ca^2+^ detection

Intracellular Ca^2+^ imaging and quantification was conducted using a Fluo-4 Ca^2+^ detection kit (Beyotime, China). Cells were incubated with Fluo-4 staining solution at 37°C for 30 min and continuously observed and photographed with a fluorescence microscope. The intracellular calcium ion concentration was calculated based on the fluorescence intensity using the Image J software.

### Intracellular reactive oxygen species detection

Cells were treated with 10 μM DCFH-DA (Beyotime, China) in a serum-free medium for 20 min and washed thrice. Subsequently, the fluorescence of DCF at 488/525 nm was measured using a fluorescence microplate reader (Bio-Tek, USA) to determine the concentration of ROS.

### Assessment of cell apoptosis rate

After trypsinization, adherent cells were collected and washed twice. They were then resuspended in 1× Binding Buffer and 100 µL of the suspension was added to the tube. It was then mixed with 5 µL of Annexin V-FITC and mixed gently. 10 µL of propyl iodide (PI) dye was added and suspension was mixed again and allowed to incubate at RT for 20 min. Annexin V-positive and PI-negative cells indicated early apoptotic cells, while both Annexin V-positive and PI-positive cells represented late apoptotic cells.

### Cell contraction assay

A collagen gel-based cell contraction assay kit (CBA-201, Cell Biolabs, USA) was utilized to compare the contraction function of primary pericytes. Briefly, the pericytes were resuspended in pericytes growth medium. The cells were mixed with a collagen gel working solution on ice. Next, collagen polymerization was induced by incubating the cell-gel combination solution (0.5 mL, containing 2 × 10^5^ cells) in a 24-well plate for 1 h at 37°C. Subsequently, the cells were treated according to the previously described groupings to assess contractile force. Each gel was continually photographed and quantified using Image J software. Cell contraction (%) is equal to (well area - gel area) / well area × 100%.

### Statistical analysis

The data were analyzed with SPSS 17.0, and expressed as mean ± standard deviation for continuous variable and as percentages (%) for categorical variable. Differences between groups were compared using the one-way ANOVA and nonparametric tests.

## Results

### IA-SCMI decreased the brain temperature

To compare the neuroprotective effect of IA-SCMI and IA-SCSI, we evaluated the cooling effect of IA-SCMI after MCAO. During the infusion, IA-SCMI gradually reduced the temperature of ipsilateral cerebral striatum and cortex to about 32°C, which was significantly lower than that of the contralateral side, and slowly warmed up after infusion. Moreover, the core temperature (rectal temperature) remained nearly unchanged (Figure 1B). The cooling effect of IA-SCMI on the ipsilateral brain was comparable to that of IA-SCSI (Figure S4A), and neither IA-SMI nor IA-SSI induced significant hypothermic effects (Figure S4B). Moreover, IA-SI including IA-SCMI did not impact vital signs, as there were no differences in MABP, heart rate, pO_2_ and pCO_2_ (Figure S5).

### IA-SCMI improved functional recovery

Having demonstrated the cooling effect of IA-SCMI, we compared the neurological behavioral protective effects between different treated groups. Following MCAO, the mNSS score and the time taken for the rats to touch and remove the tap significantly increased, and the time taken to fall off the rotating rod was shortened. Although the neural function of the MCAO group rats partially recovered after 14 days, residual neurological deficiency was still detected (Figure 1C-F). IA-SSI did not alter neurological function following MCAO (Figure 1C-F). However, based on the results of mNSS and the time taken to touch the tape, IA-SMI partially alleviated the neurological impairment (Figure 1C-E). IA-SCSI treatment significantly improved neurological function compared to MCAO, IA-SSI and IA-SMI (Figure 1C-F). Moreover, IA-SCMI further promoted the recovery of neural function (Figure 1C-F). Although no statistically significant differences were observed, IA-SMI appeared to reduce mortality compared to the MCAO and MCAO+IA-SSI groups. IA-SCSI seemed to further lower mortality, with the IA-SCMI group exhibiting the lowest mortality rate (Figure S6).

### IA-SCMI protected brain integrity

**Figure 2:**
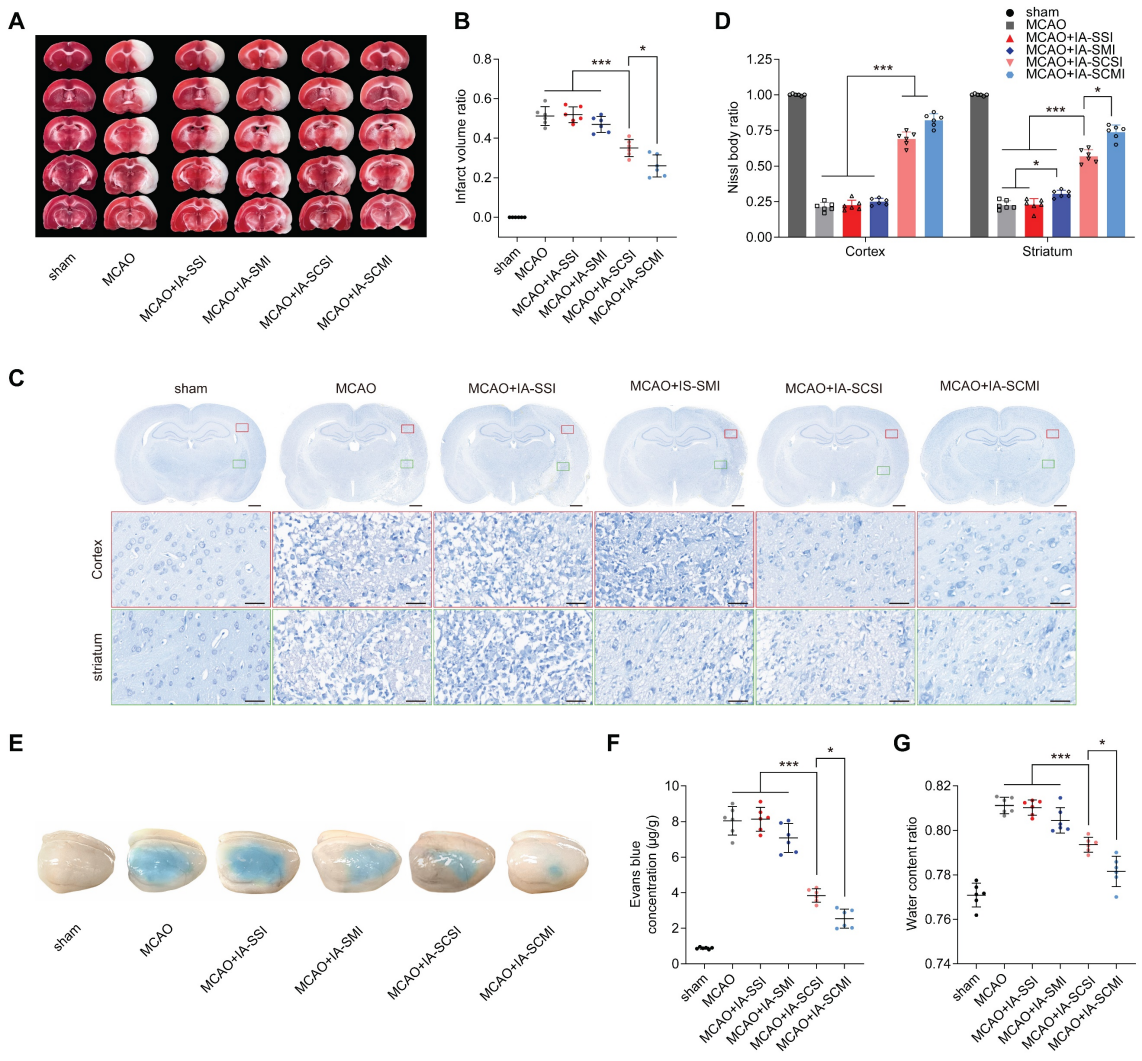
The protective effects of IA-SCMI on brain integrity. (A-B) Representative images of brain slices stained with TTC and summary data of relative infarction volumes. (C-D) Representative images of Nissl stained brain slices and summary data of relative Nissl body number. Scale bars: 1 mm and 25 µm. (E-F) Representative images of brain tissue penetrated by Evans blue and summary data of relative Evans blue content. (G) The relative water content of ipsilateral brain tissue. n=6 for each group. * *p* < 0.05, *** *p* < 0.001.

Considering the satisfactory protective effects on the neurological behavior of IA-SCMI, *ex vivo* brain integrity of neuron and BBB was further investigated. The results indicated that IA-SCSI decreased the infarction volume compared to MCAO, MCAO+IA-SSI and MCAO+IA-SMI groups. Moreover, IA-SCMI exerted a stronger protective effect than IA-SCSI (Figure 2A-B). The Evans blue leakage and brain water content test results showed a similar trend, demonstrating that IA-SCMI had the best BBB-protective effects (Figure 2E-G). Interestingly, quantitative analysis of Nissl bodies in nerve cells on the seventh day (Figure 2C-D) found that IA-SCSI and IA-SCMI conferred protection on cortical nerve cells compared to MCAO, MCAO+IA-SSI and MCAO+IA-SMI, with IA-SCMI performing relatively better compared to IA-SCSI, but the difference was not significant. Compared to MCAO and MCAO+IA-SSI, MCAO+IA-SMI showed higher protective effects on striatal neurons, with IA-SCSI having better protective effects than IA-SMI. Overall, IA-SCMI yielded the best protective effects. In summary, these results indicate that IA-SCMI can efficiently prevent brain integrity damage to the greatest extent.

### IA-SCMI enhanced CBF

**Figure 3:**
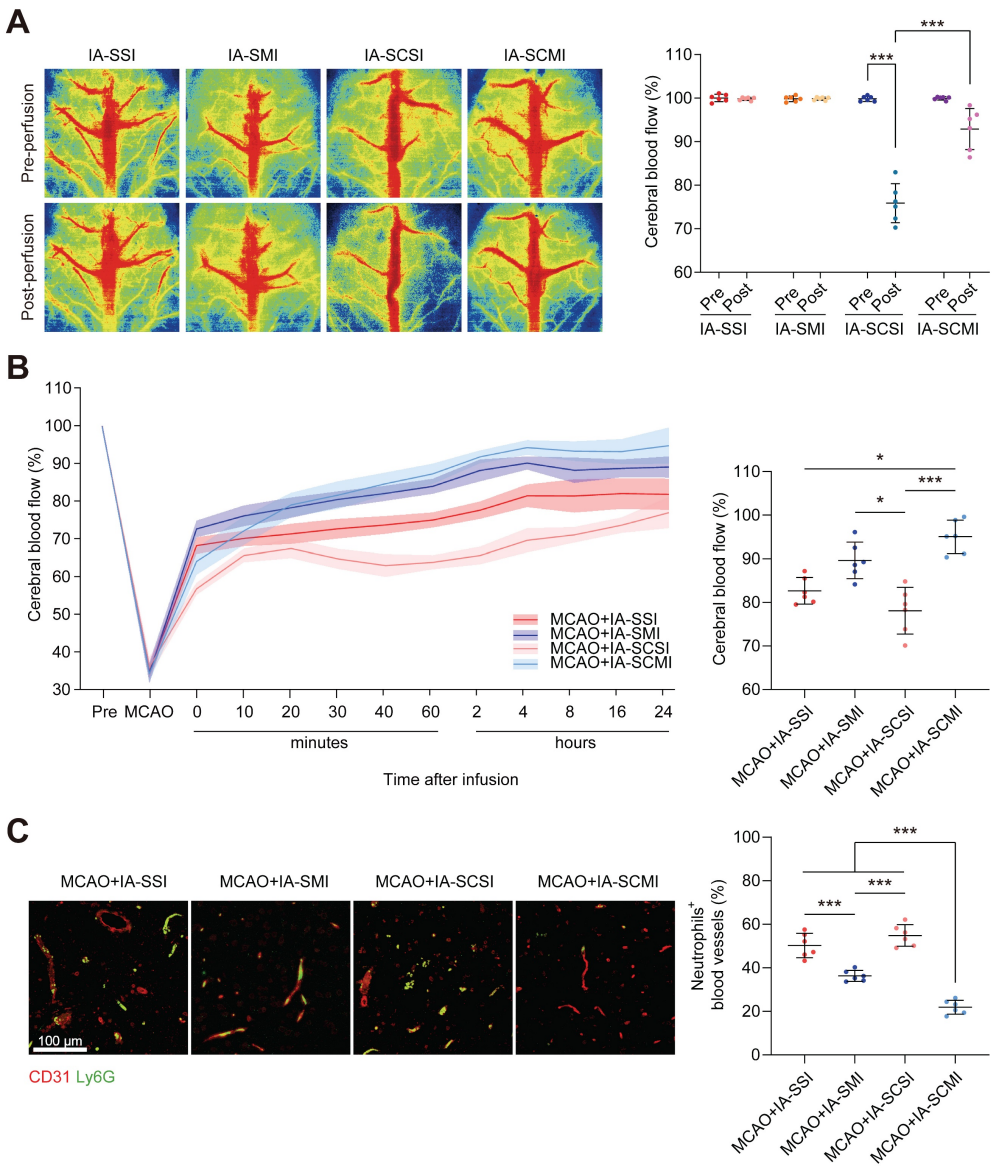
The CBF protection effect of IA-SCMI. (A) LSI image of CBF immediately after IA-SI in normal rats and summary data of related CBF. (B) Temporal profile of CBF in MCAO rats with IA-SI and summary data of the related CBF at the 24-h time point after IA-SI. (C) Fluorescence images of entrapped neutrophils (Ly6G, green) in capillaries (CD31, red) and quantification. n=6 for each group. * *p* < 0.05, *** *p* < 0.001.

Given the limited research on the impact of IA-SCSI on CBF, we initially conducted our study in normal rats. Following immediate infusion, both IA-SCSI and IA-SMI had no effect on CBF. However, IA-SCSI reduced the ipsilateral CBF, while IA-SCMI inhibited this trend (Figure 3A). Following infusion, CBF exhibited gradual recovery pattern (Figure S7), possibly due to brain temperature rewarming. Considering the transient CBF effects of IA-SI on normal rats, we further investigated the effects in MCAO rats. Figure 3B and Figure S8 show that MCAO significantly reduced ipsilateral CBF, and immediately after infusion, IA-SMI restored the CBF more effectively compared with IA-SSI, and this effect lasted up to 24 h later (not significant). However, compared to IA-SSI and IA-SMI, IA-SCSI decreased CBF immediately, and 24 h later, the CBF of the IA-SCSI group was still slightly lower than that of the IA-SSI group (not significant) and much lower than that of the IA-SMI group. Interestingly, although the CBF of IA-SCMI was lower compared with that of IA-SSI and IA-SMI immediately after infusion, it was higher than that of IA-SCSI. Further analysis revealed that CBF in IA-SCMI group was the highest among all groups 24 h after infusion. We hypothesized that the underlying reason for this phenomenon might be the delayed relaxation of microvessels during reperfusion due to hypothermia, leading to thrombus accumulation. Despite the eventual recovery of brain temperature and the long-term protective effect of hypothermia on ischemia-reperfusion injury, the early microvessel contraction and microthrombosis were irreversible, resulting in a permanent reduction in cerebral blood flow. Magnesium ions can reduce the immediate effect of hypothermia on CBF, and combined the protective effect with hypothermia late, thereby inhibit vessel contraction and microthrombosis leading to CBF enhancement. Based on this hypothesis and previous evidence that neutrophils are the main cells that block microvessels after MCAO recanalization 38, we further measured the content of neutrophils in capillaries and found that the number of neutrophils blocking in capillaries in group IA-SCSI was comparable to that of the group IA-SSI, while IA-SMI treatment significantly reduced the number of neutrophils and IA-SCMI exhibited the best inhibitory effect on neutrophil blockage (Figure 3C).

### Hypothermia combined with MgSO_4_ protected neurons and ECs *in vitro*

**Figure 4:**
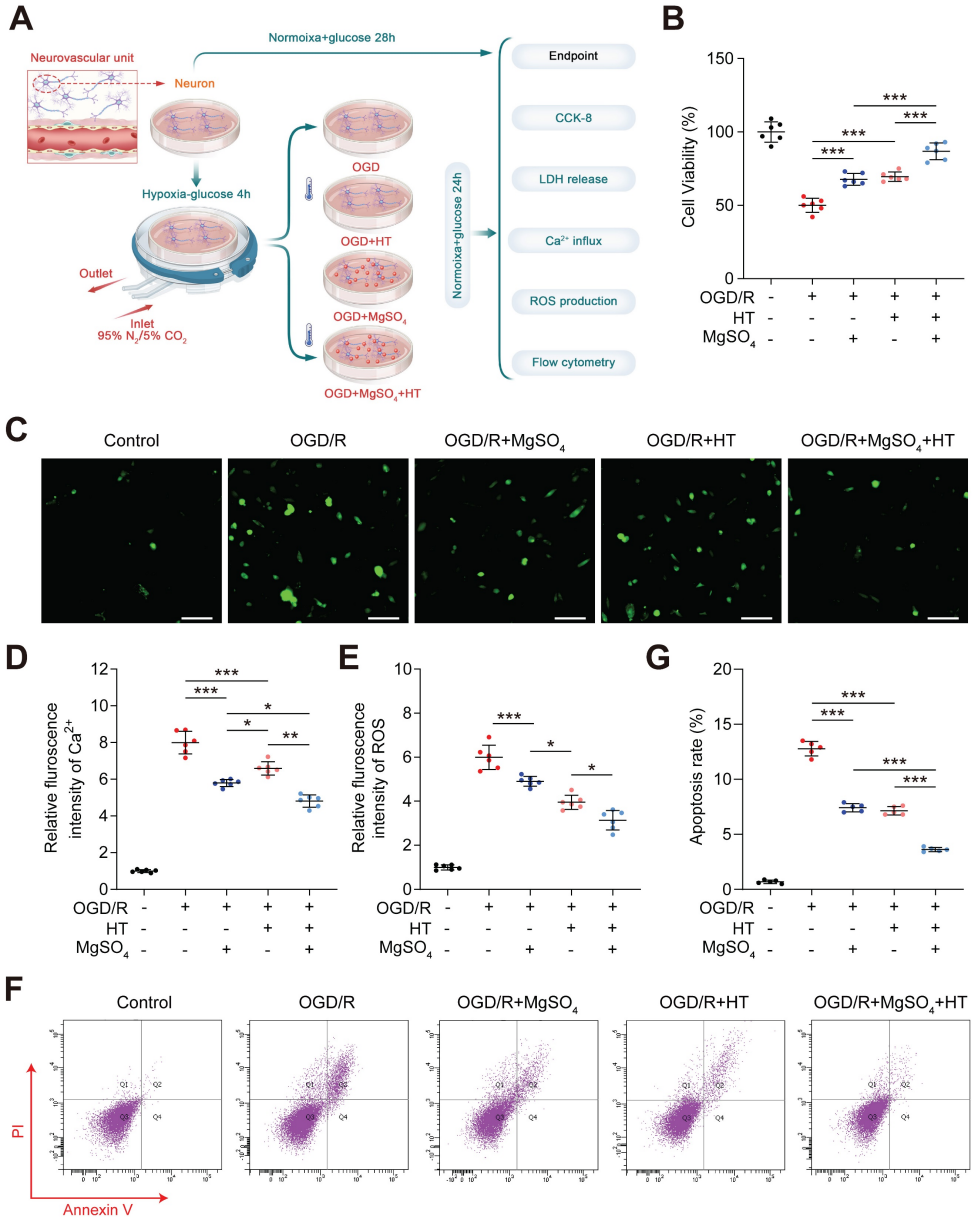
*In vitro* effects of the combination hypothermia and MgSO_4_ on neurons. (A) The flow chart of neural cell experiments. (B) Cell viability assay (n=6). (C-D) Fluorescence imaging of intracellular Ca^2+^ and summary data of relative fulroscence intensity of Ca^2+^ in neuron (n=6). scale bars: 20 µm. (E) Summary data of relative fulroscence intensity of ROS in neuron (n=6). (F-G) Flow cytometry and quantification of cell apoptosis rate (n=5). * *p* < 0.05, ** *p* < 0.01, *** *p* < 0.001.

Considering the excellent *in vivo* neuroprotective and BBB protective effects, we further investigated the synergistic effects of hypothermia and MgSO_4_ *in vitro,* which was conducted in neuron first (Figure 4A)*.* The CCK-8 assay showed that the cell viability decreased after OGD/R injury, and both hypothermia alone and MgSO_4_ alone could reverse this effect. Furthermore, the combination of hypothermia and MgSO4 demonstrated the most beneficial effect on preserving cell viability (Figure 4B). This trend was also confirmed by the LDH release assay (Figure S9A). Ca^2+^ overload and ROS production were two of the most common damaging factors for cells following OGD/R treatment. Ca^2+^ regulation may be a potential mechanism underlying the effectiveness of the combination therapy. Thus, we measured the cellular concentration of Ca^2+^ and ROS. It was observed that OGD/R increased Ca^2+^ and ROS in neuron and both MgSO_4_ alone and hypothermia alone could revers Ca^2+^ change. However, hypothermia alone reduced a larger concentration of ROS than MgSO_4_ alone. Furthermore, the combination of hypothermia and MgSO_4_ exhibited the most significant effect in reducing both Ca^2+^ and ROS (Figure 4C-E). To further elucidate the impact on cell viability, apoptosis was measured, as it is a critical factor contributing to cell death. The results indicated that the combination therapy could most effectively prevent cell apoptosis, and both hypothermia alone and MgSO_4_ alone were equally effective in reducing neural apoptosis. (Figure 4F-G).

**Figure 5:**
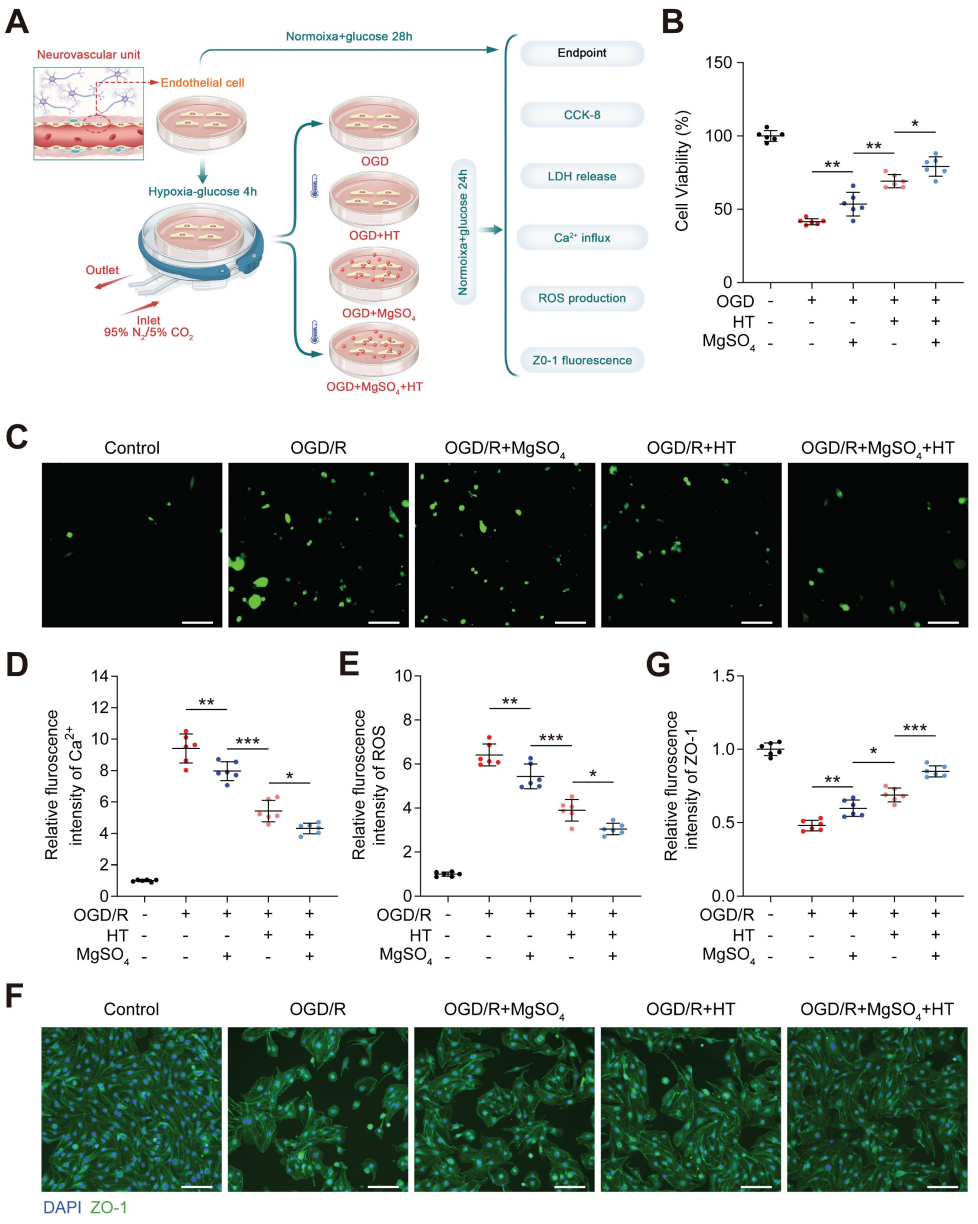
*In vitro* effects of the combination of hypothermia and MgSO_4_ on microvessel EC. (A) The flow chart of microvessel EC experiments. (B) Cell viability assay. (C-D) Fluorescence imaging of intracellular Ca^2+^ and summary data of relative fluorescence intensity of Ca^2+^ in ECs. Scale bars: 20 µm. (E) Summary data of relative fluorescence intensity of ROS in ECs. (F-G) Fluorescence imaging and quantification of cellular ZO-1 expression. n=6 for each group. * *p* < 0.05, ** *p* < 0.01, *** *p* < 0.001.

Subsequently, we investigated the combined effect of hypothermia and MgSO_4_ on microvessel ECs, an important component of BBB (Figure 5A). From the perspective of cell viability, LDH release concentration and intracellular Ca^2+^ and ROS, hypothermia combined with MgSO_4_ exhibited the best protective effect on ECs after OGD/R injury and hypothermia alone ranks second, with MgSO_4_ alone ranking last (Figure 5B-E and Figure S9B). The expression level of tight junction proteins in endothelial cells serves as a crucial marker of cell barrier function. Therefore, the expression level of ZO-1, one of the tight junction proteins, was quantified. The results indicated that OGD/R injury significantly downregulated ZO-1 expression in ECs, and MgSO_4_ alone only partially rescued ZO-1 expression. Moreover, hypothermia alone had a better salvage effect on ZO-1 reduction, whereas hypothermia combined with MgSO_4_ had the highest inhibitory effect (Figure 5F-G). These results indicated hypothermia combined with MgSO_4_ could simultaneously protect neurons and EC cells, consistent with *in vivo* data.

### MgSO_4_ counteracted the early harmful effects of hypothermia on pericytes *in vitro* to exert a synergistic role late

**Figure 6:**
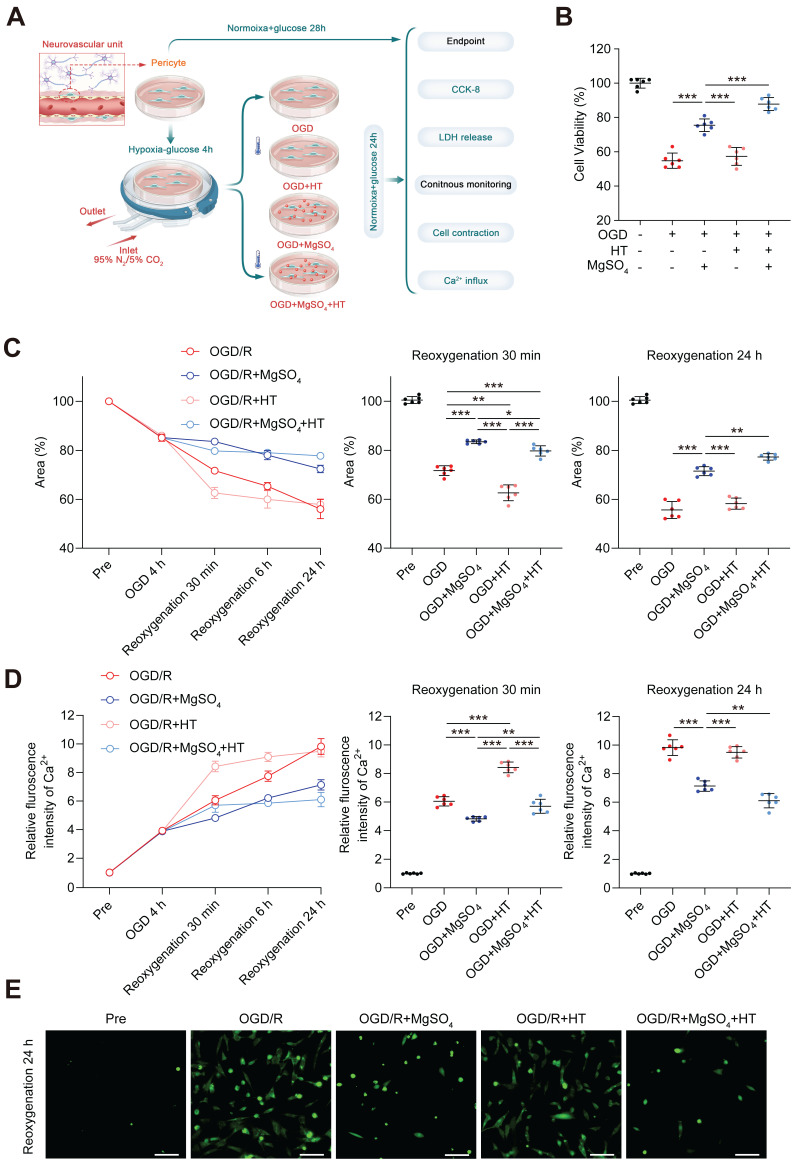
*In vitro* effects of the combination of hypothermia and MgSO_4_ on pericytes. (A) The flow chart of pericyte experiments. (B) Cell viability assay. (C) Quantitative analysis of the area of collagen gel. (D) Quantitative analysis of relative fluorescence intensity of Ca^2+^ in pericytes. (E) Fluorescence imaging of intracellular Ca^2+^ after reoxygenation 24 h. scale bars: 20 µm. n=6 for each group. * *p* < 0.05, ** *p* < 0.01, *** *p* < 0.001.

Previous studies have demonstrated that pericytes could control CBF 41. Therefore, we examined the effects of MgSO_4_ alone, hypothermia alone, and the combination of the two on pericytes *in vitro* (Figure 6A). Pericyte death has been linked to long-term capillary contraction, and therefore, we explored the survival rate of pericytes 41. Hypothermia alone did not alter the cell viability and LDH release following OGD/R injury. However, MgSO_4_ alone rescued pericyte death, and hypothermia combined with MgSO_4_ had a better protective effect (Figure 6B and Figure S9C). Next, we investigated pericyte contraction rate by examining the reduction of gel area. After OGD injury, pericyte exhibited partial contraction, and after reoxygenation, pericyte contraction increased over time up to 24 h. Moreover, exposure to hypothermia alone exacerbated the early contraction, but the final contraction rate of OGD/R+HT group was comparable with the rate of the OGD/R group. However, treatment with MgSO_4_ alone decreased early contraction and this effect lasted up to 24 h. Interestingly, the combined treatment group could counteract the early contraction-promoting effect of hypothermia alone, meanwhile, compared with the control group, there is even some protective effect. Moreover, the combination therapy yielded the highest inhibitory effect on contraction of pericytes (Figure 6C). Since Ca^2+^ influx and overload are important factors that induce pericyte death and contraction, we measured the intracellular Ca^2+^ concentration. The pattern of intracellular calcium ion increase closely resembles the pattern of pericyte contraction degree increase, except for a lack of difference in intracellular Ca^2+^ levels between the combination therapy group and the OGD/R group 30 min after reoxygenation (Figure 6D-E). Based on these results, we speculated that hypothermia alone might induce early Ca^2+^ influx into pericyte after OGD/R to cause intense premature contraction and death of pericytes, which will offset the late-stage inhibitory effect of hypothermia on Ca^2+^ influx. However, MgSO_4_ alone persistently inhibited Ca^2+^ influx to suppress the contraction and death of pericytes. Moreover, after combination therapy, MgSO_4_ abolished the early harmful effects of hypothermia on pericytes. Notably, combination therapy played a synergistic protective role in the later stages, thus achieving the best protective effect.

The mechanism of premature influx of Ca^2+^ into pericytes caused by hypothermia was further investigated in normal pericytes. Hypothermia stimulated normal pericyte contraction due to Ca^2+^ influx, while Mg^2+^, as a natural antagonist of Ca^2+^, inhibited this phenomenon (Figure S10). The *in vitro* and *in vivo* results indicated that the contraction effect of hypothermia and Mg^2+^ on pericytes maybe an inherent phenomenon that is not affected by I/R and OGD/R injury. Previous studies have reported that TRPM8, a cold-sensitive channel, is expressed on smooth muscle cells of cerebral blood vessels from piglets. TRPM8 regulates Ca^2+^ influx into cells by sensing cold stimuli 42. Therefore, TRPM8 may mediate the aforementioned effects. Given that TRPM8 has not been reported in cerebral pericytes of rats, its expression was explored in this study, and the effects of hypothermia and MgSO_4_ on TRPM8 were also investigated. Cellular immunofluorescence showed that TRPM8 was expressed on rat cerebral pericytes (Figure S11). The effects of hypothermia and TRPM8 agonist, menthol, on the contraction and Ca^2+^ influx of pericytes were inhibited by the TRPM8 antagonist, AMTB, and MgSO_4_, respectively (Figure S12). These findings suggest that the early Ca^2+^ influx effect of mild hypothermia on pericytes is mediated by the activation of the TRPM8 channel, while MgSO_4_ can antagonize this channel, thereby inhibiting the effects of hypothermia.

## Discussion

**Figure 7:**
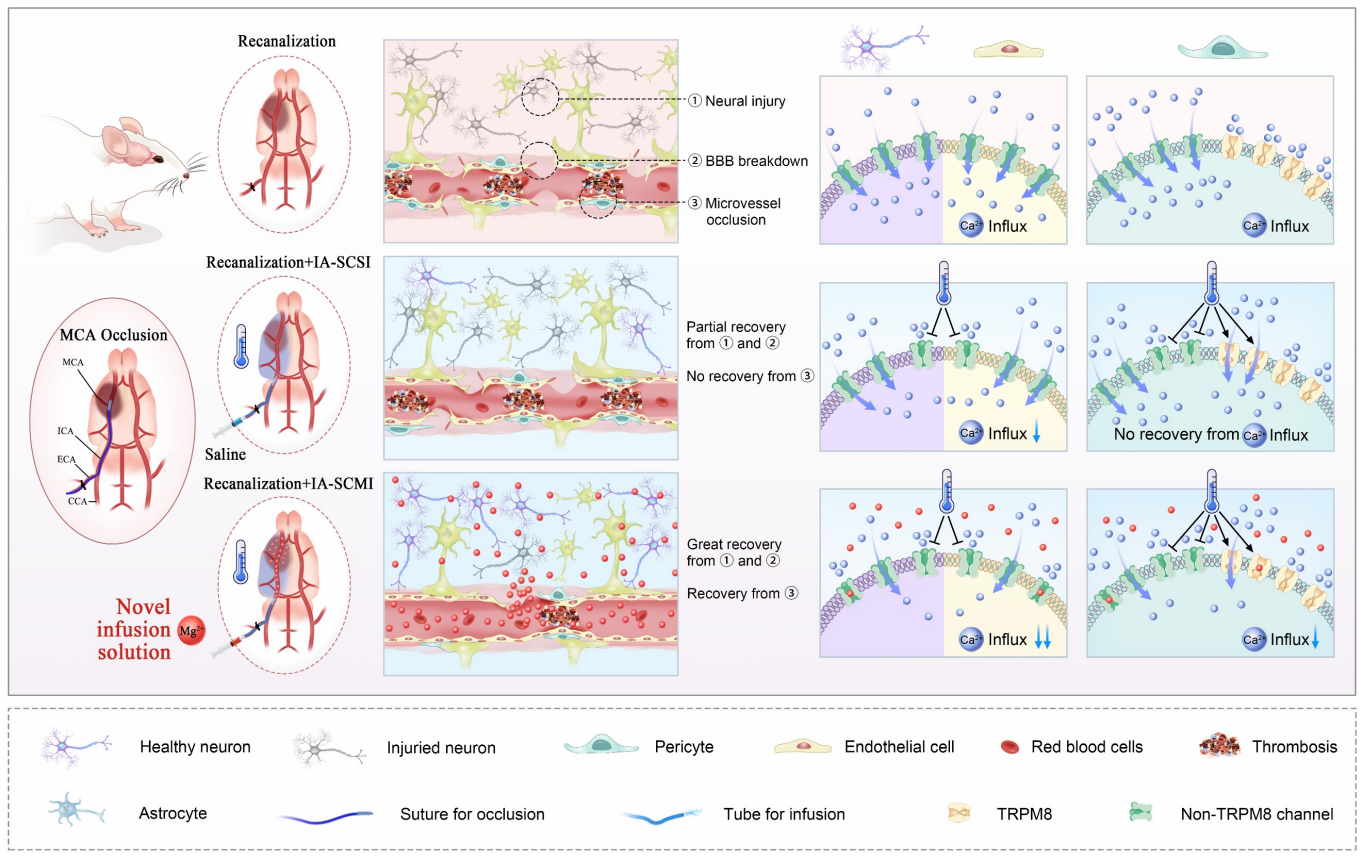
MgSO_4_ as a novel hypothermia infusion solution for promoting AIS recovery by regulating Ca^2+^ in NVUs. After I/R injury, the infarction region undergoes severe neural injury, BBB breakdown and microvessel occlusion, which were triggered by Ca^2+^ influx into neurons, cerebral microvessel ECs and pericytes. IA-SCSI cooled the brain and promoted partial recovery from neural injury and BBB breakdown but could not rescue microvessel occlusion. *In vitro* cell experiments confirmed that hypothermia could partially inhibit Ca^2+^ influx into neurons and cerebral microvessel ECs but did not affect the total amount of calcium ions in pericytes because hypothermia increased Ca^2+^ influx through TRPM8. Moreover, IA-SCMI demonstrated significant recovery from neural injury and BBB breakdown, and enhanced microvessel circulation. The combination of hypothermia and Mg^2+^ exerted stronger inhibitory effects on Ca^2+^ influx compared with hypothermia alone in NVU cells. MCA, middle cerebral artery; ICA, internal carotid artery; ECA, external carotid artery; CCA, common carotid artery; IA-SCSI, intra-artery selective cooling saline infusion; IA-SCMI, intra-artery selective cooling MgSO_4_ infusion; BBB, brain-blood barrier.

This study explored the possibility of MgSO_4_ as a novel hypothermia infusion solution for treating AIS. The result showed that IA-SCMI selectively cooled infarction ipsilateral brain tissue, and compared to IA-SCSI and IA-SMI, IA-SCMI more efficiently reduced neurological deficits. Moreover, IA-SCMI improved neuron viability and BBB integrity. In contrast, IA-SCSI had no beneficial effects on CBF, but IA-SCMI abolished the early CBF reduction induced by hypothermia and improved CBF recovery. Furthermore, the combination of hypothermia and Mg^2+^ protected neurons and cerebral microvessel ECs by inhibiting Ca^2+^ influx *in vitro*. *In vitro* pericyte experiments revealed that hypothermia had no protective effect on pericyte survival and promoted early rapid contraction through the activation of TRPM8. In contrast, combination therapy could counteract the early detrimental effects of hypothermia and synergistically inhibited Ca^2+^ influx into pericytes, promoting their viability and inhibiting their contraction (Figure 7).

### Innovation points of IA-SCMI

Compared to the existing simple hypothermia therapies for AIS, such as whole-body cooling, ice cap cooling, and IA-SCSI, IA-SCMI encompasses the effects of targeted brain hypothermia and targeted drug therapy through arterial infusion pathway. In the present study, we found that IA-SCMI achieved superior therapeutic effects compared to IA-SCSI. Therefore, the innovative findings of our research indicate that monotherapy may not be sufficiently effective for AIS. Instead, AIS should be treated with a combination of targeted physical (hypothermia) and pharmacological (MgSO_4_) therapies, akin to the approach used in some cancer treatments (e.g., targeted radiotherapy plus chemotherapy), in order to achieve the best possible outcomes. This innovation points also provides ideas for future IA-SI research, which can use cooling solutions containing different neuroprotective drugs as infusion media to better treat AIS. Another innovation of this study is the exploitation of the Ca^2+^ antagonistic effect of MgSO_4_ to potentiate the protective effects of hypothermia on neuron and ECs, and reverses the adverse effects on CBF. Future studies should leverage this novel mechanism to select drugs that can achieve synergistic therapy based on hypothermia.

### Hypothermia combined with Mg^2+^ for treating AIS

The combination of hypothermia and Mg^2+^ has shown potential to treat AIS in many previous studies. For example, both Zausinger *et al.* 16 and Campbell *et al.* 17 have found that the combination of intravenous administration of Mg^2+^ and surface cooling is more effective in treating AIS than either treatment alone. Their results strongly support the findings of the present research. Moreover, intraarterial infusion Mg^2+^ into infarction region can amplify the neuroprotective effect than intravenous infusion 43, and compare to surface cooling, IA-SCI has faster and stronger cooling effect 10. Therefore, IA-SCMI has great potential to achieve excellent therapeutic effects and our results confirmed this potential.

### Hypothermia combined with Mg^2+^ for treating other cerebral infarction disease

Besides AIS, several other cerebral infarction diseases such as hypoxic-ischemic encephalopathy in newborns and cardiac arrest, have been studied to examine whether they can benefit from combination therapy of hypothermia and Mg^2+^. For instance, Zhu *et al.* found that combined therapy was more effective at reducing CA1 neuronal death than either treatment used alone in global cerebral ischemic rats 44. In clinical practice, hypothermia is the only effective treatment for patients with neonatal hypoxic-ischemic encephalopathy and the combination of hypothermia and MgSO_4_ has also been widely tested in clinical practice. Rahman *et al.* conducted a pilot study to primarily validate the safety of combination therapy 45. These results provide indirect support for the efficiency and safety of combination therapy.

### The combined protective effects of hypothermia and Mg^2+^ on nerves and BBB

Numerous studies have demonstrated the protective effect of hypothermia on neural injury and BBB breakdown 46, 47. Similarly, Mg^2+^ was reported to attenuate neural death and BBB damage 48, 49. Therefore, the combined application of hypothermia and Mg^2+^ may have synergistic protective effects on nerves and BBB. Although this combination therapy decreased the infarct size and promote neurological recovery, it is not known whether it can improve the integrity of BBB, and the protective mechanism first discovered in this study has not been thoroughly explored. Magnesium ions, as a naturally powerful Ca^2+^ antagonist, could conquer Ca^2+^ overload 19. Moreover, hypothermia plays a multiple role in Ca^2+^ influx, such as through inhibiting oxidative stress damage and stabilizing mitochondrial function 50. Thus, Ca^2+^ regulation seem to be the potential mechanism for combination therapy. The hypothesis was confirmed through *in vitro* experiments. Although the effects of hypothermia alone and Mg^2+^ alone on Ca^2+^ influx in neurons and ECs varied, the combination of these two exhibited the best protective effect in both types of cells. Moreover, studies have shown that combination therapy more efficiently inhibit ROS and cell death. Therefore, such a combination will inhibit neuronal apoptosis and loss of tight junction proteins in ECs.

### The combined regulation effects of hypothermia and Mg^2+^ on CBF

Temperature influences the blood flow is a common phenomenon in whole body and previous research has demonstrated that CBF can also be modulated by focal temperature 15. This result aligns with our findings, suggesting that CBF can be stably suppressed under hypothermic conditions. This phenomenon partially explains why CBF may not fully recover after reperfusion following hypothermia. However, Mg^2+^ can counteract this early adverse effect, suggesting that hypothermia and Mg^2+^ both regulate CBF by modulating Ca^2+^ influx into certain cells. CBF is mainly controlled by capillary diameter, and pericytes are the main regulators of capillary diameter 41. TRPM8, a cold sensitive Ca^2+^ influx channel, has been detected in cerebral vessel and pericyte within tumors 42, 51. The regulatory effects of hypothermia and Mg^2+^ on capillary diameter may be mediated by Ca^2+^ influx through TRPM8 on pericytes, which influences intracellular Ca^2+^ concentration and regulates their contraction state. The *in vitro* pericyte experiments in this study partially support this hypothesis, providing an explanation for the observed phenomenon *in vivo*. Future studies should aim to provide more direct *in vivo* evidence for this mechanism.

### The regulation effect of Mg^2+^ on Ca^2+^ homeostasis in different NVU cells

Mg^2+^ and Ca^2+^ are both divalent ions with similar chemical properties, suggesting that Mg^2+^ may compete with Ca^2+^ for the same non-selective cation channel on cerebral cells *in vivo,* generating antagonistic effects. Recent studies have provided some clues as to which channel Mg^2+^ act on in each NVU cell.

Several kinds of Ca^2+^ channels are expressed on the cell membranes of neurons. The Ca^2+^ overload caused by glutamate mediated N-methyl-D-aspartic acid (NMDA) receptor activation has been found to be the main mechanism contributing to neuronal I/R injury, and the blocking effect of Mg^2+^ on this channel was documented nearly 40 years ago 52, 53. In recent years, with the discovery of the transient receptor potential (TRP) family channel proteins that regulate Ca^2+^ penetration, studies have found that the TRP melastatin 7 (TRPM7) may be activated *in vitro* by oxidative stress injury, which then induces neuronal death due to the occurrence of Ca^2+^ overload. In contrast, downregulation of TRPM7 in hippocampal neurons *in vivo* prevented I/R injury-induced neuronal death 54, 55. It has also been reported that Mg^2+^ can block TRPM7 to reduce Ca^2+^ influx, thereby inhibiting the damage caused by oxidative stress 56. Based on these findings, we speculated that the protective effect of Mg^2+^ may involve inhibition of the neuronal damage caused by TRPM7-triggered Ca^2+^ overload.

Currently, few studies have explored the regulation of Ca^2+^ overload in cerebral microvessel ECs following AIS. In a recent study, it was found that TPPM2 influenced the occurrence of Ca^2+^ overload in microvessel ECs after I/R injury, implying that BBB breakdown, neurological function deterioration, and inhibition of TRPM2 may rescue this adverse effect 57. Moreover, another study showed that TRPM2 exhibited similar permeability for Mg^2+^ and Ca^2+^ 58. Thus, the inhibitory effect of Mg^2+^ on Ca^2+^ overload in cerebral microvessel ECs identified in our study may be mediated by the blocking effect of Mg^2+^ on TRPM2.

Voltage-gated Ca^2+^ channels (VGCC) are the main channels for controlling the intracellular Ca^2+^ concentration and contraction of microvessel diameter 59. As the main regulator of cerebral microvascular diameter, the contraction of pericytes is also primarily regulated by VGCC. For example, a recent study showed that the L-type VGCC of cerebral pericytes was activated after I/R injury via the TMEM16A-mediated Cl^-^ influx and the subsequent cell membrane depolarization, leading to pericyte contraction and cerebral “no-flow” phenomenon 60. Mg^2+^ can also inhibit VGCC 61, so we speculate that the inhibitory effect of Mg^2+^ on pericyte contraction after I/R injury is mediated by VGCC on pericytes.

In summary, based on our results and previous research, we infer that Mg^2+^, as a natural antagonist of Ca^2+^, participates in Ca^2+^ homeostasis in different NVU cells following I/R injury, however, considering that the main Ca^2+^ regulatory pathways in each cell are different, the exact sites of action for Mg^2+^ may also be inconsistent. In the future, well-designed experiments are advocated to investigate the regulation of Ca^2+^ homeostasis in NVU cells in the context of I/R injury and explore the effects of Mg^2+^ on its regulatory pathways.

### Perspectives

This study shows that MgSO_4_ is a novel hypothermia infusion solution for promoting ischemic stroke recovery in rats. In the future, we hope to validate our findings in larger animals. Considering that hypothermia and MgSO_4_ are common treatment methods, the combination therapy can be directly performed in clinical practice. Owing to the recent development of another new IA-SCI method using special instruments, IA-selective cooling autologous blood infusion (IA-SCAI) is expected to become a new approach for achieving targeted brain cooling 62, 63. This technology can effectively address the challenge of long-term inability to maintain arterial cooling infusion due to capacity limitations. In the future, Mg^2+^ could be added to the perfused cooling blood to potentially enhance its neuroprotective effects.

Mechanistically, diverse protective pathways are involved in the regulation of hypothermia and Mg^2+^ effects. However, this study only explored selected superficial mechanisms, especially those associated with Ca^2+^ in NVU cells. Further comprehensive and in-depth investigations are advocated.

## Conclusion

In summary, this study used MgSO_4_ as a novel hypothermia infusion solution to assist in the synergistic inhibition of nerve and BBB damage, and reverse the early inhibitory effect of hypothermia on CBF, thereby synergistically protecting eventual CBF. Different Ca^2+^ regulatory pathways were identified in various NVU cells, including neuron, mcirovessel ECs and pericytes, as possible mechanisms of the combination therapy.

## Supplementary Material

Supplementary figures and table.

## Abbreviations

AIS: acute ischemic stroke
BBB: blood-brain barrier
CBF: cerebral blood reflow
CCA: common carotid artery
CCK-8: cell counting kit-8
DMEM: Dulbecco's modified Eagle's medium
EB: Evans blue
ECA: external carotid artery
ECs: endothelial cells
FBS: fetal bovine serum
IA-SCAI: intra-arterial selective cooling autologous blood infusion
IA-SCMI: intra-arterial selective cooling infusion solution
IA-SCSI: intra-arterial selective cooling saline infusion
IA-SI: intra-arterial selective infusion
ICA: internal carotid artery
I/R: ischemia/reperfusion
LDH: lactic dehydrogenase
MABP: mean arterial blood pressure
MCAO: middle cerebral artery occlusion
MgCl_2_: magnesium chloride
MgSO_4_: magnesium sulfate
mNSS: modified neuropathy symptom score
NMDA: N-methyl-D-aspartic acid
NVUs: neurovascular units
OGD/R: oxygen-glucose deprivation/reoxygenation
PBS: phosphate-buffered saline
pCO_2_: arterial carbon dioxide partial pressure
PI: propyl iodide
pO_2_: arterial oxygen partial pressure
RT: room temperature
SD: Sprague-Dawley
TRP: transient receptor potential
TRPM7: transient receptor potential melastatin 7
TTC: triphenyltetrazolium chloride
VGCC: Voltage-gated Ca^2+^ channels
