# Supplementary material for: MgSO4 as a novel hypothermia infusion solution promotes ischemic stroke recovery through Ca2+ regulation of neurovascular units
Source: Theranostics. 2025 Jan 2;15(5):1896–913. doi: 10.7150/thno.104879 (PMC11780535; doi:10.7150/thno.104879)
Supplement: Supplementary file 1 — Supplementary figures and table. [file thnov15p1896s1.pdf]

Supplementary Materials for

$MgSO_4$ as a novel hypothermia infusion solution promotes ischemic stroke recovery through Ca^{2+} regulation of neurovascular units

Yang Zhang *et al.*

*Corresponding author: Xunming Ji (jixm@ccmu.edu.cn); Ming Li (liming@xwhosp.org);
Yufeng Zheng (yfzheng@pku.edu.cn)

This PDF file includes:

Table S1;

Figure S1 to S12.

Table S1. The modified neurological severity score (mNSS) scoring standards.

Scoring sections	Scoring Standards	Points
**Motor tests**	Raising the rat by the tail	3
	Flexion of the forelimb	1
	Flexion of the hindlimb	1
	Head moving > 10° to vertical axis within 30 s	1
	Placing the rat on the floor	3
	Normal walk	0
	Inability to walk straight	1
	Circling toward paretic side	2
	Falling down to paretic side	3
**Sensory tests**	Limb placing test (moving the rat laterally toward the table)	
	Reaching the table slowly with limbs or could not move at all	1
	Proprioceptive test (pushing the paw against table edge to stimulate limb muscles)	
	Losing the resistance	1
**Balance tests**	Beam balance tests (2.5 cm wide)	6
	Balancing with steady posture	0
	Grasping side of the beam	1
	Hugging the beam and 1 limb falling down from the beam	2
	Hugging the beam and 2 limbs falling down from beam, or spins on beam (> 60 s)	3
	Attempting to balance on the beam but falling off (> 40 s)	4
	Attempting to balance on the beam but falling off (> 20 s)	5
	Falls off; no attempt to balance or hang on to beam (< 20 s)	6
**Reflexes**	Touching the auditory meatus	
	Not shaking the head	1
	Slightly touching the cornea with cotton	
	No eye blink	1
	Making a brief noise	
	No motor response	1
**Abnormal movements**	Seizures, myoclonus, or myodystony	1
**Maximum points**		18

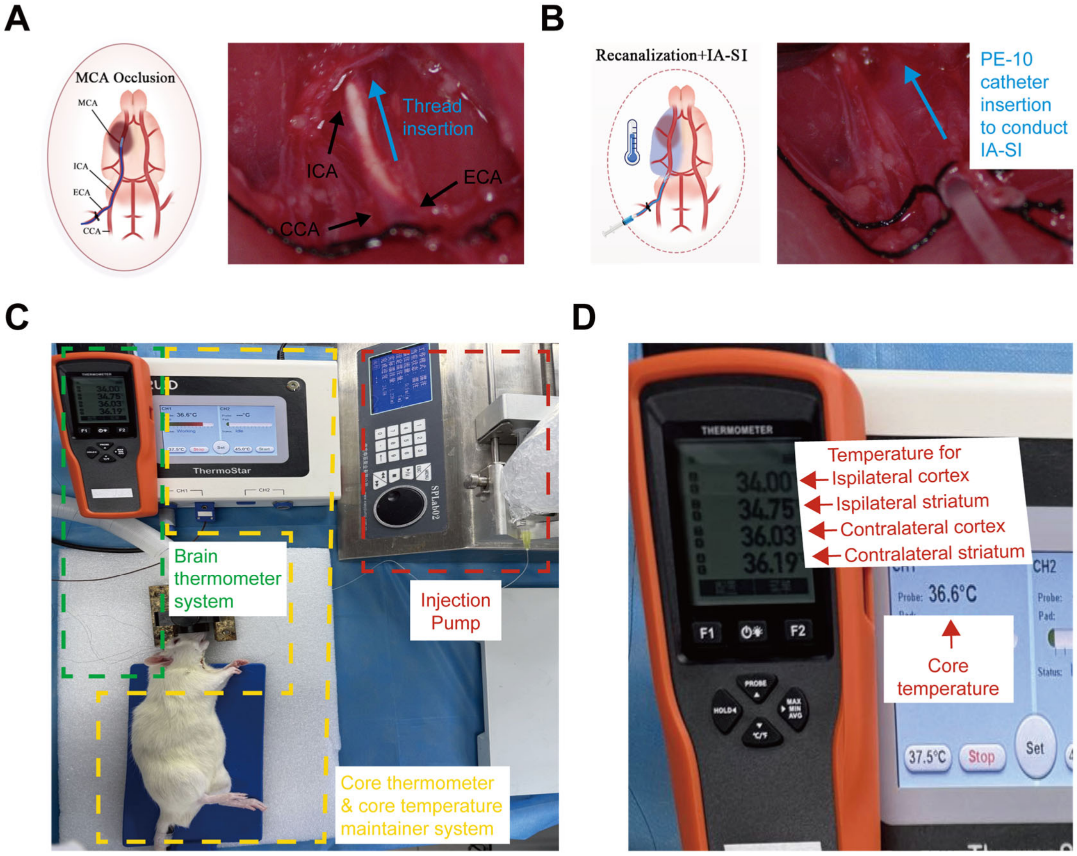

Figure S1. The detailed process for transient middle cerebral artery occlussion (MCAO), intra-arterial selective infusion (IA-SI), techniques used to maintain the body and measure the brain temperature throughout the experiment. (A) MCAO modeling process: a thread was inserted from external carotid artery (ECA) to internal carotid artery (ICA). CCA, common carotid artery. (B) IA-SI process: a PE-10 catheter was inserted from ECA to ICA to infuse solution to brain through artery. (C) Techniques used to infuse solution, maintain and measure the body temperature, and measure brain temperature: the PE-10 catheter was connected to a syringe placed in the injection pump for constant speed infusion; the core temperature was monitored by inserting temperature-sensor wires into the rectum, and to maintain core temperature, rats were placed on a feedback-regulated heating pad; the temperature-sensor wires connected to the thermometer were inserted into the ipsilateral and contralateral cerebral cortex and striatum of rats to measure brain temperature. (D) Amplification of thermometer readings for brain temperature and core temperature in (C).

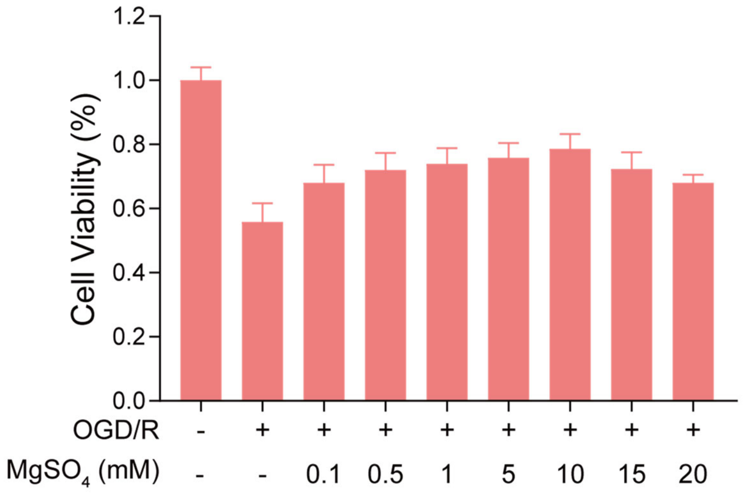

Figure S2. The neuroprotective effect of gradient $MgSO_4$ concentrations was evaluated through cell viability. (n=5 for each group)

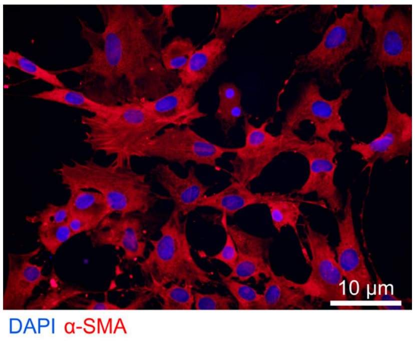

Figure S3. Fluorescence image for identification of primary cultured pericytes.

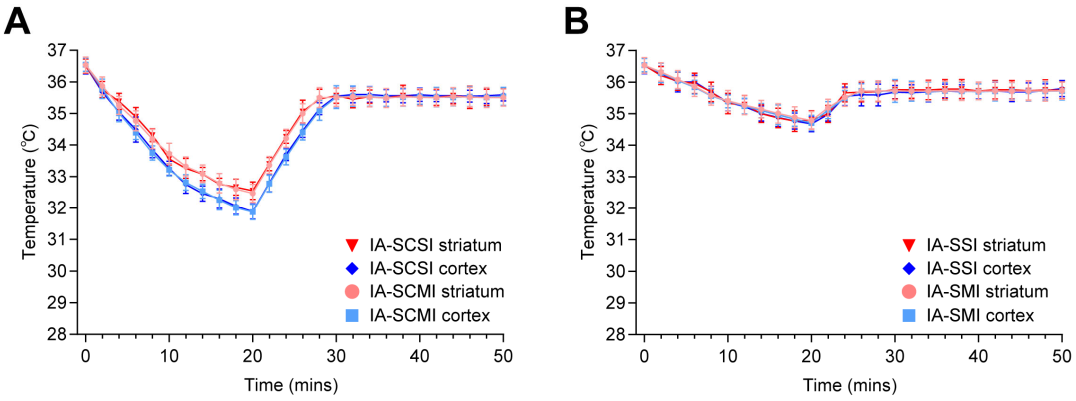

Figure S4. The cooling effect on ipsilateral striatum and cortex of (A) intra-arterial selective cooling saline and $MgSO_4$ infusion (IA-SCSI and IA-SCMI), and (B) intra-arterial selective saline and $MgSO_4$ infusion (IA-SSI and IA- SMI). (n=5 for each group)

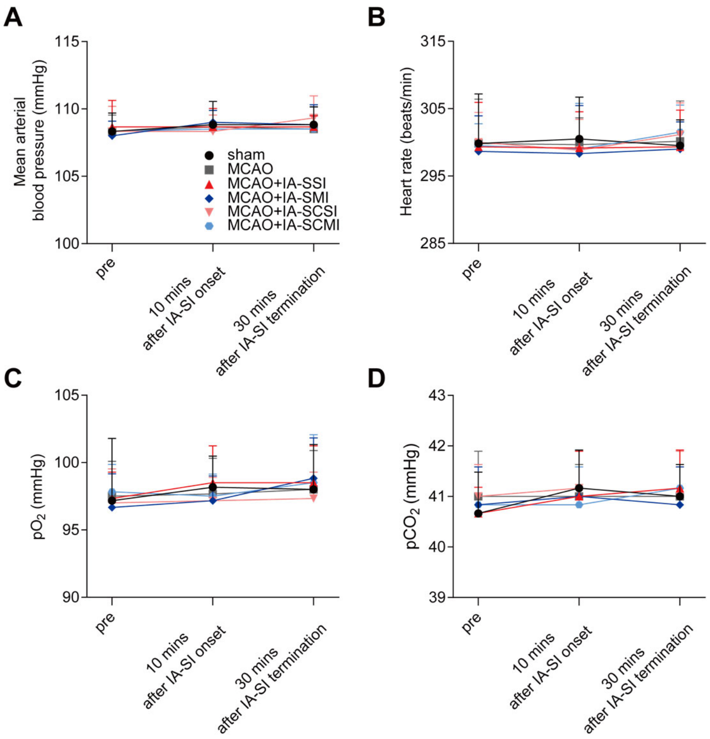

Figure S5. Physiology parameters before, during and after IA-SI. (A) Mean arterial blood pressure. (B) Heart rate. (C-D) pO_2 and pCO_2. (n=6 for each group)

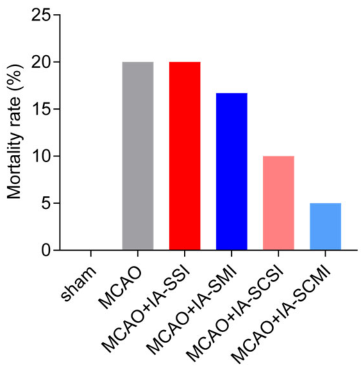

Figure S6. Mortality rate of rats after MCAO and IA-SI.

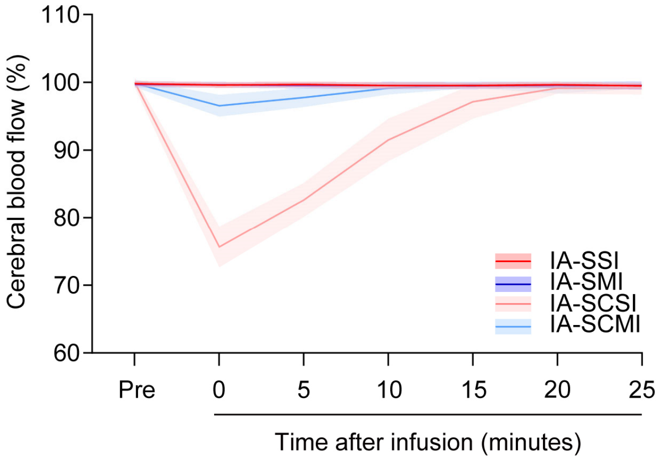

Figure S7. Temporal profile of CBF after IA-SI in normal rats. (n=6 for each group)

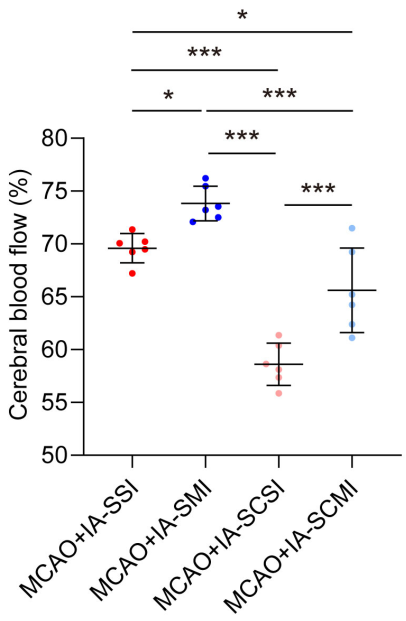

Figure S8. Summary data of relative CBF immediately after IA-SI in MCAO rats. (n=6 for each group)

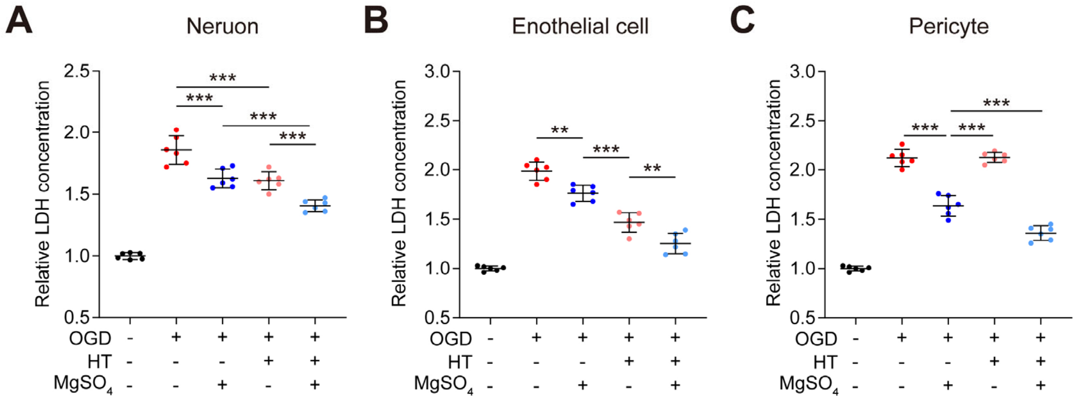

Figure S9. Relative LDH release concentration of (A) neuron, (B) endothelial cell and (C) pericyte. (n=6 for each group)

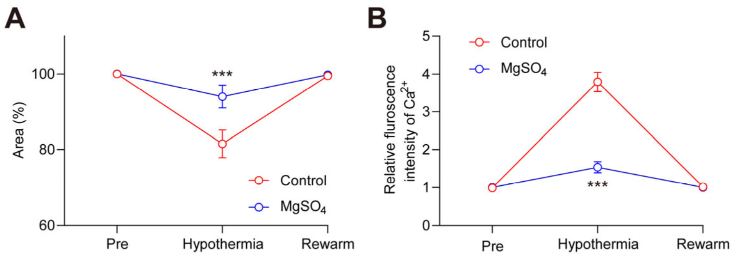

Figure S10. (A) Comparison of the contractile pattern of normal pericytes treated with hypothermia in the absence or presence of $MgSO_4$. (B) Quantitative analysis of relative fluorescence intensity of Ca^{2+} in pericytes. (n=6 for each group)

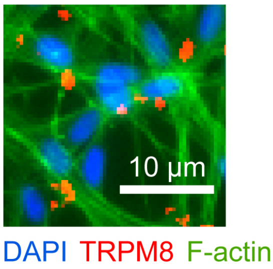

Figure S11. Fluorescence imaging of TRPM8 in pericytes.

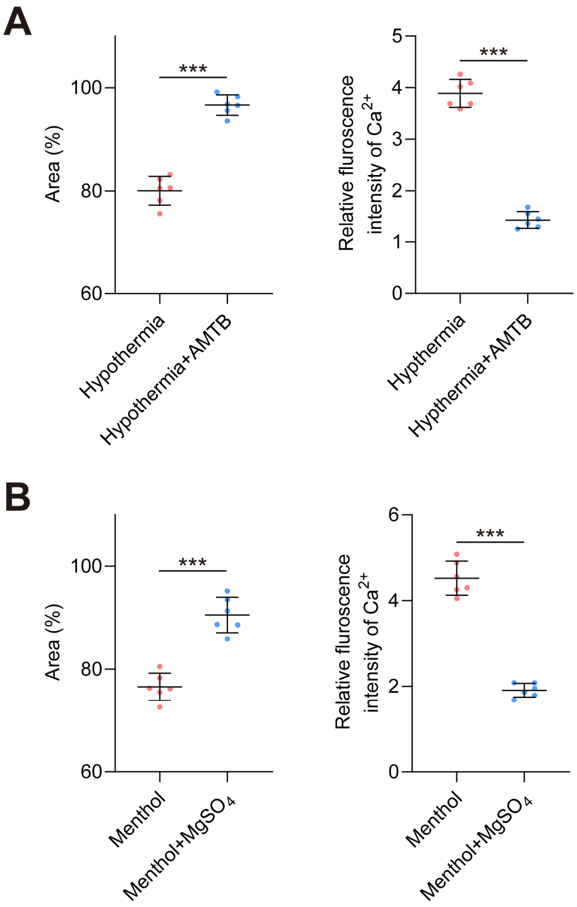

Figure S12. Comparison of the contractile and Ca^{2+} influx degree of normal pericytes (A) treated with hypothermia in the absence or presence of AMTB, and (B) treated with menthol in the absence or presence of $MgSO_4$. (n=6 for each group)
